# Supplementary material for: Single cell transcriptional evolution of myeloid leukemia of Down syndrome
Source: Nat Commun. 2026 Apr 23;17:3474. doi: 10.1038/s41467-026-71707-2 (PMC13106683; doi:10.1038/s41467-026-71707-2)
Supplement: Supplementary file 1 — Supplementary Information [file 41467_2026_71707_MOESM1_ESM.pdf]

Single cell transcriptional evolution of myeloid leukaemia of Down syndrome

Authors: Mi K. Trinh[1], Konstantin Schuschel[2,3], Hasan Issa[2,3], Rebecca Thomas[4], Conor Parks[1], Agnes Oszlanczi[1], Toochi Ogbonnah[1], Di Zhou[1], Lira Mamanova[1], Elena Prigmore[1], Emilia R. Robertson[4], Angus Hodder[1,4], Anna Wenger[1], Nathaniel D. Anderson[1], Holly J. Whitfield[1], Taryn D. Treger[1,5,6], José Gonçalves-Dias[2,3], Karin Straathof[4,7,8], David O'Connor[4,9], Matthew D. Young[1], Laura Jardine[1,10], Stuart Adams[4], Jan-Henning Klusmann[2,3,11]*, Jack Bartram[4,9]*, Sam Behjati[1,4,5,6]*

Affiliations:

[1]Wellcome Sanger Institute; Hinxton, CB10 1SA, UK.

[2]Department of Pediatrics, Goethe University Frankfurt, Frankfurt, Germany.

[3]Frankfurt Cancer Institute, Frankfurt am Main, Germany.

[4]Great Ormond Street Hospital for Children NHS Foundation Trust, London, WC1N 3JH, UK.

[5]Department of Paediatrics, University of Cambridge, Cambridge, CB2 0QQ, UK.

[6]Cambridge University Hospitals NHS Foundation Trust, Cambridge, CB2 0QQ, UK.

[7]UCL Cancer Institute, 72 Huntley St, London, WC1E 6DD, UK.

[8]Great Ormond Street Biomedical Research Centre, 30 Guilford Street, London, WC1N 1EH, UK.

[9]UCL Great Ormond Street Institute of Child Health; London, WC1N 1EH, UK.

[10]Biosciences Institute, Newcastle University; Newcastle upon Tyne, NE2 4HH, UK.

[11]German Cancer Consortium (DKTK), Partner Site Frankfurt/Mainz and German Cancer Research Center (DKFZ), Heidelberg, Germany.

*Co-corresponding and co-directing authors

Contact information: klusmann@em.uni-frankfurt.de; Jack.Bartram@gosh.nhs.uk; sb31@sanger.ac.uk

Supplementary Information

Supplementary Figures

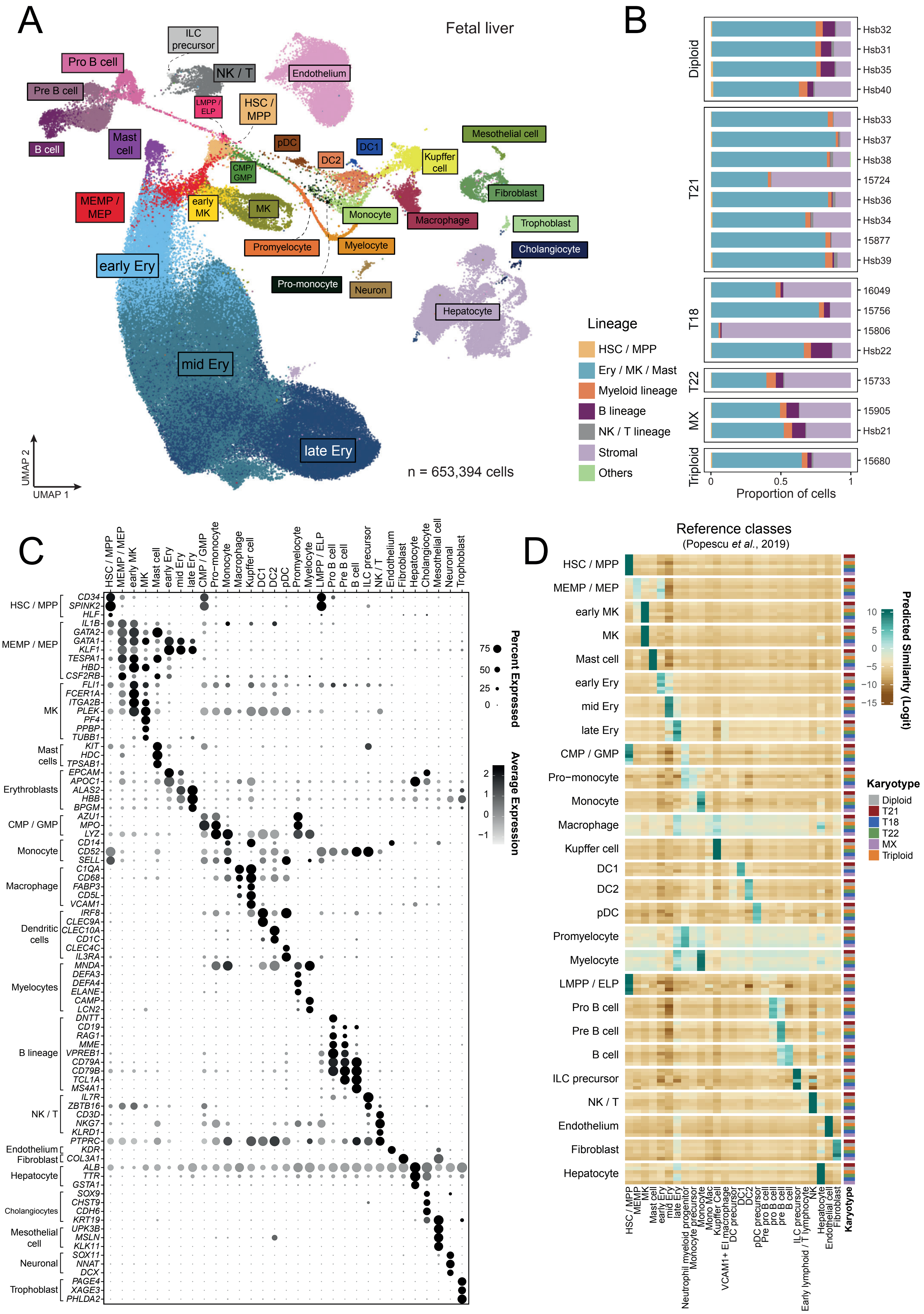
A
Fetal liver
ILC precursor
NK / T
Endothelium
Pro B cell
Pre B cell
LMPP / ELP
HSC / MPP
B cell
Mast cell
pDC
DC1
Mesothelial cell
DC2
Kupffer cell
CMP / GMP
MEMP / MEP
early MK
MK
Monocyte
Macrophage
Fibroblast
Trophoblast
Promyelocyte
Myelocyte
Cholangiocyte
early Ery
Pro-monocyte
Neuron
Hepatocyte
mid Ery
late Ery
UMAP 2
UMAP 1
n = 653,394 cells
B
Diploid
T21
T18
T22
MX
Triploid
Hsb32
Hsb31
Hsb35
Hsb40
Hsb33
Hsb37
Hsb38
15724
Hsb36
Hsb34
15877
Hsb39
16049
15756
15806
Hsb22
15733
15905
Hsb21
15680
0
0.5
1
Proportion of cells
Lineage
HSC / MPP
Ery / MK / Mast
Myeloid lineage
B lineage
NK / T lineage
Stromal
Others
C
HSC / MPP
MEMP / MEP
early MK
MK
Mast cell
early Ery
mid Ery
late Ery
CMP / GMP
Pro−monocyte
Monocyte
Macrophage
Kupffer cell
DC1
DC2
pDC
Promyelocyte
Myelocyte
LMPP / ELP
Pro B cell
Pre B cell
B cell
ILC precursor
NK / T
Endothelium
Fibroblast
Hepatocyte
Cholangiocyte
Mesothelial cell
Neuronal
Trophoblast
HSC / MPP: CD34, SPINK2, HLF
MEMP / MEP: IL1B, GATA2, GATA1, KLF1, TESPA1, HBD, CSF2RB
MK: FLI1, FCER1A, ITGA2B, PLEK, PF4, PPBP, TUBB1
Mast cells: KIT, HDC, TPSAB1
Erythroblasts: EPCAM, APOC1, ALAS2, HBB, BPGM
CMP / GMP: AZU1, MPO, LYZ
Monocyte: CD14, CD52, SELL
Macrophage: C1QA, CD68, FABP3, CD5L, VCAM1
Dendritic cells: IRF8, CLEC9A, CLEC10A, CD1C, CLEC4C, IL3RA
Myelocytes: MNDA, DEFA3, DEFA4, ELANE, CAMP, LCN2
B lineage: DNTT, CD19, RAG1, MME, VPREB1, CD79A, CD79B, TCL1A, MS4A1
NK / T: IL7R, ZBTB16, CD3D, NKG7, KLRD1, PTPRC
Endothelium: KDR
Fibroblast: COL3A1
Hepatocyte: ALB, TTR, GSTA1
Cholangiocytes: SOX9, CHST9, CDH6, KRT19
Mesothelial cell: UPK3B, MSLN, KLK11
Neuronal: SOX11, NNAT, DCX
Trophoblast: PAGE4, XAGE3, PHLDA2
Percent Expressed
75
50
25
0
Average Expression
2
1
0
−1
D
Reference classes
(Popescu et al., 2019)
HSC / MPP
MEMP / MEP
early MK
MK
Mast cell
early Ery
mid Ery
late Ery
CMP / GMP
Pro−monocyte
Monocyte
Macrophage
Kupffer cell
DC1
DC2
pDC
Promyelocyte
Myelocyte
LMPP / ELP
Pro B cell
Pre B cell
B cell
ILC precursor
NK / T
Endothelium
Fibroblast
Hepatocyte
Predicted Similarity (Logit)
10
5
0
−5
−10
−15
Karyotype
Diploid
T21
T18
T22
MX
Triploid
HSC / MPP
MEMP
MK
Mast cell
early Ery
mid Ery
late Ery
Neutrophil myeloid progenitor
Monocyte precursor
Monocyte
Mono Mac
Kupffer Cell
VCAM1+ EI macrophage
DC precursor
DC1
DC2
pDC precursor
Pre pro B cell
pro B cell
pre B cell
B cell
ILC precursor
Early lymphoid / T lymphocyte
NK
Hepatocyte
Endothelial cell
Fibroblast
Karyotype

Supplementary Figure 1: Fetal liver scRNA-seq dataset.

(A) Uniform Manifold Approximation and Projection (UMAP) visualisation of the fetal liver scRNA-seq dataset (detailed in Table 1, Supplementary Data 1), where cells (dots) are coloured by cell types.

(B) Bar plot showing the proportions of different lineages captured from each fetal liver sample.

(C) Dot plot showing the z-scaled mean expression levels (colour) of key cell-type defining marker genes. Dot size represents the proportion of cells within each category with positive expression.

(D) Heatmap showing the average predicted similarity score (calculated using a CellTypist[1] logistic regression model) for each query cell type from our fetal liver scRNA-seq dataset (row panels) compared to reference cell types from the published cellular fetal liver atlas by Popescu, DM., *et al.*, 2019[2] (columns). Within each query cell type, cells are further grouped based on their respective karyotypes (individual rows). Darker green indicates stronger similarity, darker brown indicates stronger dissimilarity.

Abbreviation

Cell types: CMP / GMP - common myeloid progenitor / granulocyte-monocyte progenitor;
DC - dendritic cell; Ery - erythroblast; HSC / MPP - haematopoietic stem cell / multipotent progenitor; ILC precursor - innate lymphoid cell precursor;
LMPP / ELP - lymphoid-primed multipotent progenitor / early lymphoid progenitor;
MEMP/MEP - megakaryocyte-erythroid-mast cell progenitor / megakaryocyte-erythroid progenitor;
MK - megakaryocyte; Mono Mac - monocyte / macrophage; NK / T - natural killer cell / T cell; pDC - plasmacytoid dendritic cell; VCAM1+ EI macrophage - VCAM1+ erythroblastic island macrophages.
Karyotypes: T - trisomy; MX - monosomy X.

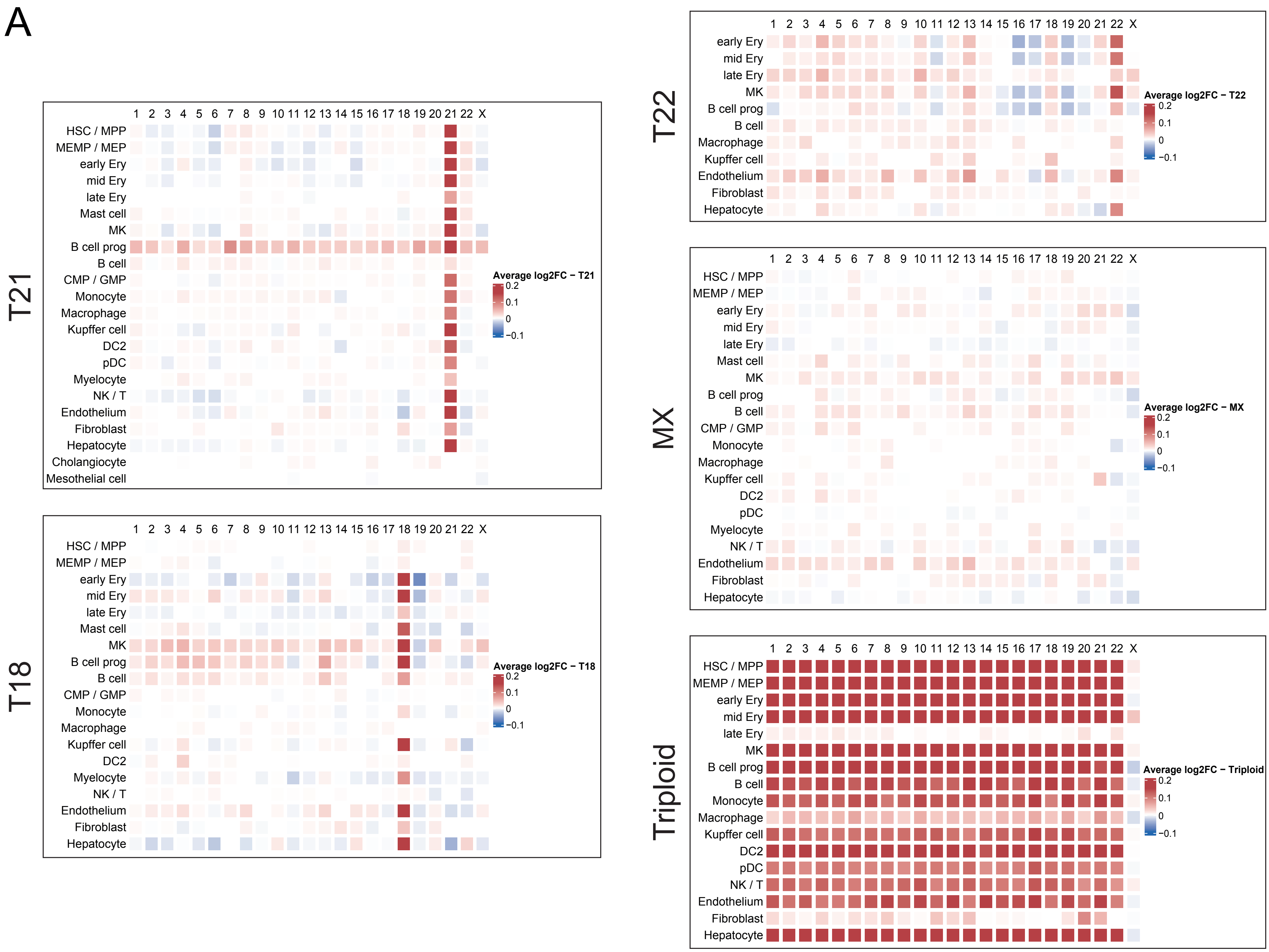
A
T21
T18
T22
MX
Triploid
1 2 3 4 5 6 7 8 9 10 11 12 13 14 15 16 17 18 19 20 21 22 X
HSC / MPP
MEMP / MEP
early Ery
mid Ery
late Ery
Mast cell
MK
B cell prog
B cell
CMP / GMP
Monocyte
Macrophage
Kupffer cell
DC2
pDC
Myelocyte
NK / T
Endothelium
Fibroblast
Hepatocyte
Cholangiocyte
Mesothelial cell
Average log2FC − T21
Average log2FC − T18
Average log2FC − T22
Average log2FC − MX
Average log2FC − Triploid
0.2
0.1
0
−0.1

Supplementary Figure 2: Transcriptional consequences of abnormal karyotypes across fetal hepatic cell types

(A) Per-chromosome median log2 fold change of gene expression levels when comparing each abnormal karyotype against the corresponding diploid counterpart for each cell type. Darker red indicates higher expression level in cells with abnormal karyotype, darker blue indicates decreased expression in cells with abnormal karyotype. “B cell prog” includes progenitors of B-cell lineage: LMPP / ELP, pro B cell, and pre B cell; “NK / T” includes ILC precursors and NK / T cells.

Abbreviation

Cell types: CMP / GMP - common myeloid progenitor / granulocyte-monocyte progenitor;
DC - dendritic cell; Ery - erythroblast; HSC / MPP - haematopoietic stem cell / multipotent progenitor;
LMPP / ELP - lymphoid-primed multipotent progenitor / early lymphoid progenitor;
MEMP/MEP - megakaryocyte-erythroid-mast cell progenitor / megakaryocyte-erythroid progenitor;
MK - megakaryocyte; NK / T - natural killer cell / T cell; pDC - plasmacytoid dendritic cell.
Karyotypes: T - trisomy; MX - monosomy X.

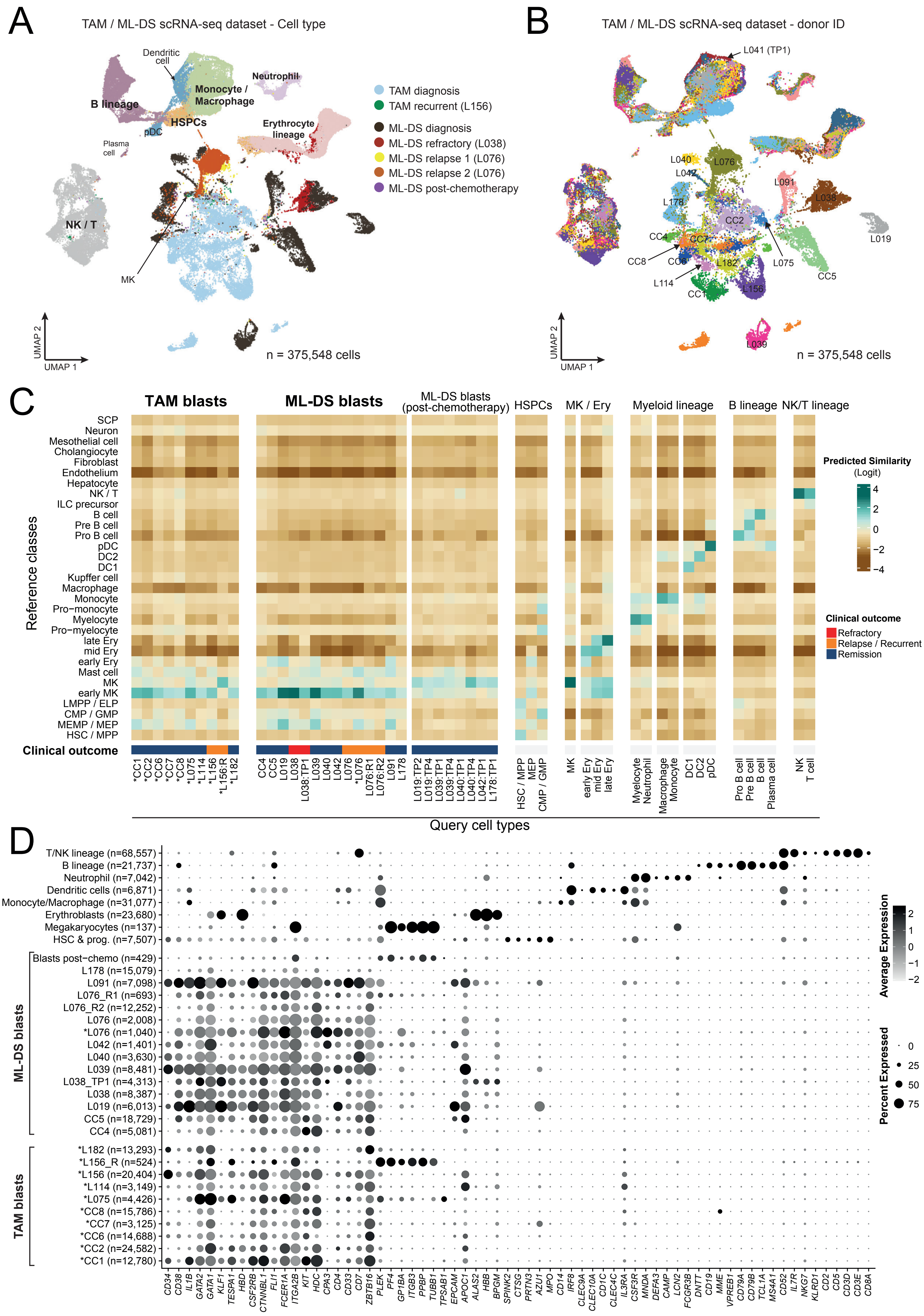
A
TAM / ML-DS scRNA-seq dataset - Cell type
Dendritic cell
Neutrophil
Monocyte / Macrophage
B lineage
HSPCs
pDC
Erythrocyte lineage
Plasma cell
NK / T
MK
UMAP 2
UMAP 1
n = 375,548 cells
TAM diagnosis
TAM recurrent (L156)
ML-DS diagnosis
ML-DS refractory (L038)
ML-DS relapse 1 (L076)
ML-DS relapse 2 (L076)
ML-DS post-chemotherapy
B
TAM / ML-DS scRNA-seq dataset - donor ID
L041 (TP1)
L040
L076
L042
L091
L038
L178
CC2
CC4
CC7
L075
L019
CC8
CC6
L182
CC5
L114
CC1
L156
L039
UMAP 2
UMAP 1
n = 375,548 cells
C
TAM blasts
ML-DS blasts
ML-DS blasts (post-chemotherapy)
HSPCs
MK / Ery
Myeloid lineage
B lineage
NK/T lineage
Reference classes
SCP
Neuron
Mesothelial cell
Cholangiocyte
Fibroblast
Endothelium
Hepatocyte
NK / T
ILC precursor
B cell
Pre B cell
Pro B cell
pDC
DC2
DC1
Kupffer cell
Macrophage
Monocyte
Pro−monocyte
Myelocyte
Pro−myelocyte
late Ery
mid Ery
early Ery
Mast cell
MK
early MK
LMPP / ELP
CMP / GMP
MEMP / MEP
HSC / MPP
Clinical outcome
*CC1
*CC2
*CC6
*CC7
*CC8
*L075
*L114
*L156
*L156:R
*L182
CC4
CC5
L019
L038
L038:TP1
L039
L040
L042
L076
*L076
L076:R1
L076:R2
L091
L178
L019:TP2
L019:TP4
L039:TP1
L039:TP4
L040:TP1
L040:TP4
L042:TP1
L178:TP1
HSC / MPP
MEP
CMP / GMP
MK
early Ery
mid Ery
late Ery
Myelocyte
Neutrophil
Macrophage
Monocyte
DC1
DC2
pDC
Pro B cell
Pre B cell
B cell
Plasma cell
NK
T cell
Query cell types
Predicted Similarity (Logit)
4
2
0
−2
−4
Clinical outcome
Refractory
Relapse / Recurrent
Remission
D
T/NK lineage (n=68,557)
B lineage (n=21,737)
Neutrophil (n=7,042)
Dendritic cells (n=6,871)
Monocyte/Macrophage (n=31,077)
Erythroblasts (n=23,680)
Megakaryocytes (n=137)
HSC & prog. (n=7,507)
ML-DS blasts
Blasts post−chemo (n=429)
L178 (n=15,079)
L091 (n=7,098)
L076_R1 (n=693)
L076_R2 (n=12,252)
L076 (n=2,008)
*L076 (n=1,040)
L042 (n=1,401)
L040 (n=3,630)
L039 (n=8,481)
L038_TP1 (n=4,313)
L038 (n=8,387)
L019 (n=6,013)
CC5 (n=18,729)
CC4 (n=5,081)
TAM blasts
*L182 (n=13,293)
*L156_R (n=524)
*L156 (n=20,404)
*L114 (n=3,149)
*L075 (n=4,426)
*CC8 (n=15,786)
*CC7 (n=3,125)
*CC6 (n=14,688)
*CC2 (n=24,582)
*CC1 (n=12,780)
CD34
CD38
IL1B
GATA2
GATA1
KLF1
TESPA1
HBD
CSF2RB
CTNNBL1
FLI1
FCER1A
ITGA2B
KIT
HDC
CPA3
CD4
CD33
CD7
ZBTB16
PLEK
PF4
GP1BA
ITGB3
PPBP
TUBB1
TPSAB1
EPCAM
APOC1
ALAS2
HBB
BPGM
SPINK2
CTSG
PRTN3
AZU1
MPO
CD14
IRF8
CLEC9A
CLEC10A
CD1C
CLEC4C
IL3RA
CSF3R
MNDA
DEFA3
CAMP
LCN2
FCGR3B
DNTT
CD19
MME
VPREB1
CD79A
CD79B
TCL1A
MS4A1
CD52
IL7R
NKG7
KLRD1
CD2
CD5
CD3D
CD3E
CD8A
Average Expression
2
1
0
−1
−2
Percent Expressed
0
25
50
75

Supplementary Figure 3: TAM / ML-DS scRNA-seq dataset.

(A) UMAP visualisation of the TAM / ML-DS scRNA-seq dataset (detailed in Table 1, Supplementary Data 1), where cells (dots) are coloured by their corresponding cell types.

(B) UMAP visualisation of the TAM / ML-DS scRNA-seq dataset, where cells (dots) are coloured by the corresponding donor ID. Leukaemic blasts generally form donor-specific clusters, whereas normal cells across donors are clustered by cell type identities.

(C) Heatmap showing the average predicted similarity score (calculated by a logistic regression model) for each query cell type from our TAM / ML-DS scRNA-seq dataset (column panels) compared to reference cell types from our fetal liver scRNA-seq dataset (rows). Darker green indicates stronger similarity, darker brown indicates stronger dissimilarity. Leukaemic cells are grouped by patient / timepoint / tissue. Samples are collected at initial diagnosis (treatment naive), unless indicated otherwise in the group name. Asterisks (*) indicate peripheral blood samples, all others are bone marrow aspirates. Leukaemic cells broadly resemble cell types along the erythrocyte-megakaryocyte-mast cell lineage, with the strongest similarity against the early megakaryocyte signal. Cell types from the normal compartment generally strongly match the corresponding reference classes.

(D) Dot plot showing the z-scaled mean expression levels (colour) of key cell type – defining marker genes. Dot size represents the proportion of cells within each category with positive expression. Normal cells are grouped into their corresponding lineages as shown in (C). Leukaemic blasts are grouped by patient / timepoint / tissue, as detailed in (C). The number of cells in each category is indicated inside brackets. Asterisks (*) indicate peripheral blood samples; all others are bone marrow aspirates.

Abbreviation

Cell types: HSPCs - haematopoietic stem and progenitor cells;
CMP / GMP - common myeloid progenitor / granulocyte-monocyte progenitor; DC - dendritic cell;
Ery - erythroblast; HSC / MPP - haematopoietic stem cell / multipotent progenitor;
ILC precursor - innate lymphoid cell precursor;
LMPP / ELP - lymphoid-primed multipotent progenitor / early lymphoid progenitor;
MEMP / MEP - megakaryocyte-erythroid-mast cell progenitor / megakaryocyte-erythroid progenitor;
MK - megakaryocyte; NK / T - natural killer cell / T cell; pDC - plasmacytoid dendritic cell;
SCP - Schwann cell precursor.
Sample timepoint: R - recurrent; R1 - relapse 1 diagnosis; R2 - relapse 2 diagnosis;
TP1 - timepoint 1, TP2 - timepoint 2, TP4 - timepoint 4.

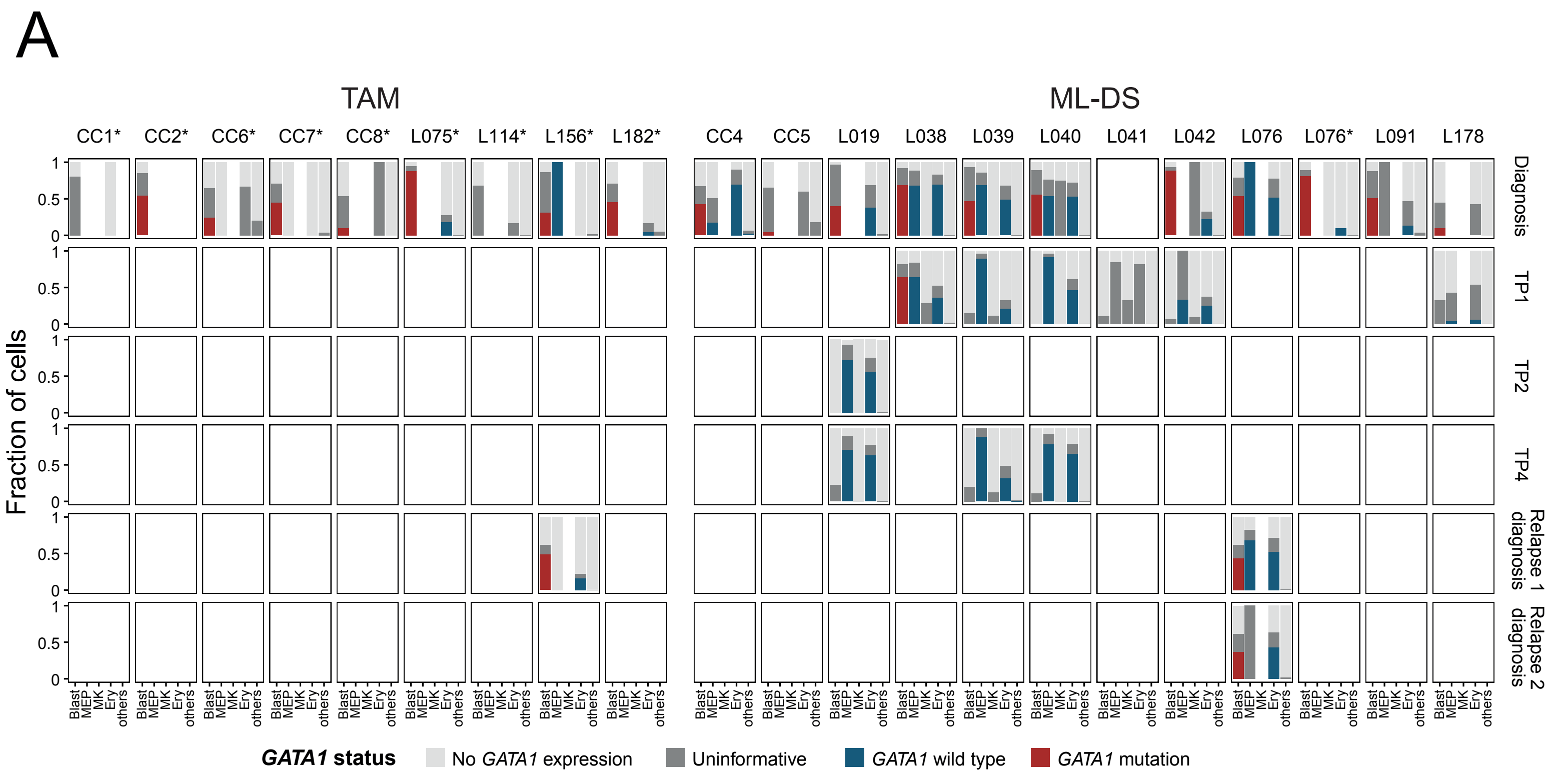

A
TAM
ML-DS
CC1*
CC2*
CC6*
CC7*
CC8*
L075*
L114*
L156*
L182*
CC4
CC5
L019
L038
L039
L040
L041
L042
L076
L076*
L091
L178
Diagnosis
TP1
TP2
TP4
Relapse 1 diagnosis
Relapse 2 diagnosis
Fraction of cells
0
0.5
1
Blast
MEP
MK
Ery
others
GATA1 status
No GATA1 expression
Uninformative
GATA1 wild type
GATA1 mutation

Supplementary Figure 4: Single cell genotyping for *GATA1* mutation.

(A) Bar plots showing the proportion of cells from each patient and timepoint categorised by *GATA1* genotypes. TAM/ML-DS blasts and cells along the megakaryocyte-erythroid-mast lineage are shown as individual columns; all other cells are grouped as "others" category. *GATA1* mutations are only detected in the blast populations. Asterisks (*) indicate peripheral blood samples; all others are bone marrow aspirates.

Abbreviation

<u>Sample timepoint</u>: TP1 - timepoint 1, TP2 - timepoint 2, TP4 - timepoint 4.

A

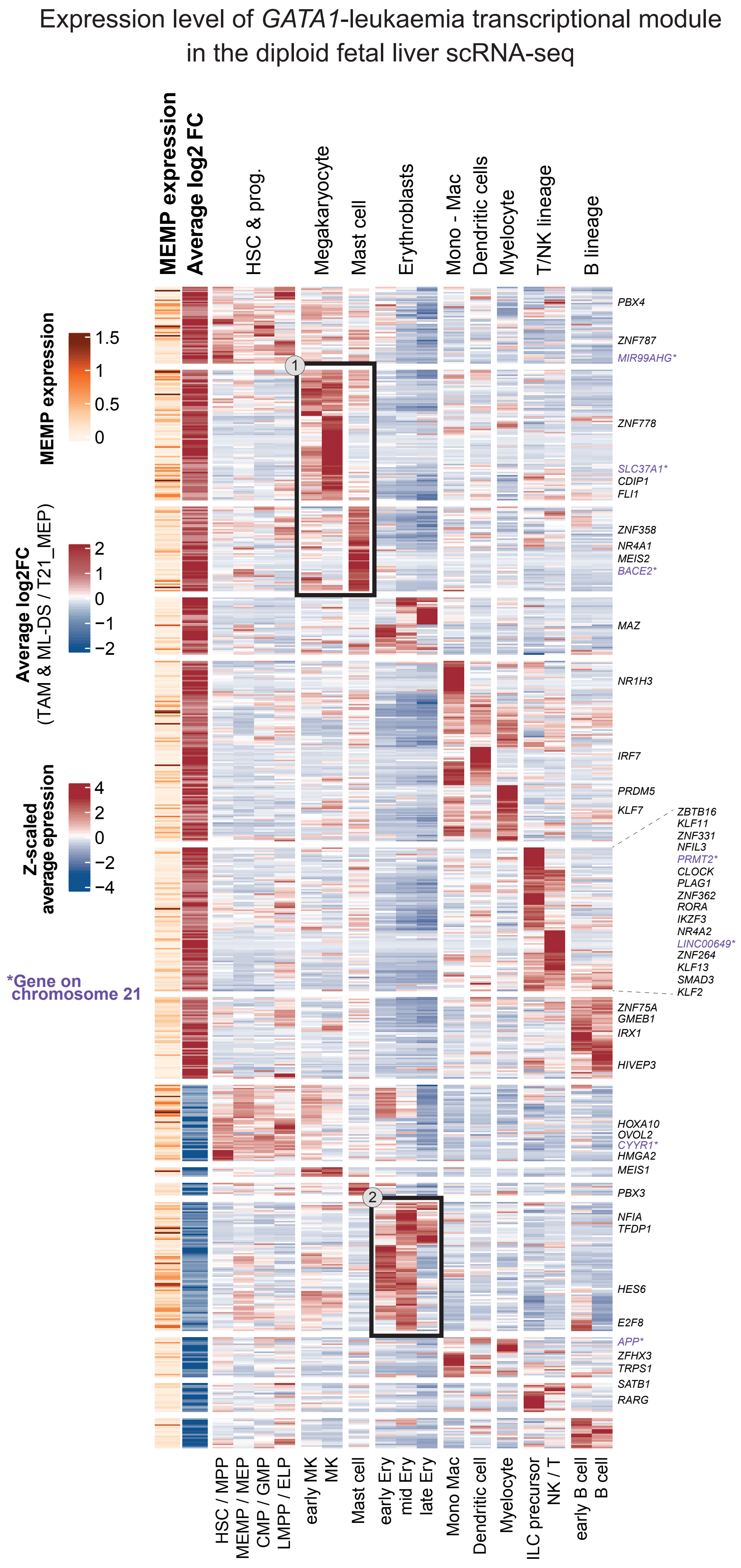
Expression level of GATA1-leukaemia transcriptional module
in the diploid fetal liver scRNA-seq
MEMP expression
Average log2 FC
HSC & prog.
Megakaryocyte
Mast cell
Erythroblasts
Mono - Mac
Dendritic cells
Myelocyte
T/NK lineage
B lineage
MEMP expression
1.5
1
0.5
0
Average log2FC
(TAM & ML-DS / T21_MEP)
2
1
0
−1
−2
Z-scaled
average epression
4
2
0
−2
−4
*Gene on chromosome 21
PBX4
ZNF787
MIR99AHG*
ZNF778
SLC37A1*
CDIP1
FLI1
ZNF358
NR4A1
MEIS2
BACE2*
MAZ
NR1H3
IRF7
PRDM5
KLF7
ZBTB16
KLF11
ZNF331
NFIL3
PRMT2*
CLOCK
PLAG1
ZNF362
RORA
IKZF3
NR4A2
LINC00649*
ZNF264
KLF13
SMAD3
KLF2
ZNF75A
GMEB1
IRX1
HIVEP3
HOXA10
OVOL2
CYYR1*
HMGA2
MEIS1
PBX3
NFIA
TFDP1
HES6
E2F8
APP*
ZFHX3
TRPS1
SATB1
RARG
1
2
HSC / MPP
MEMP / MEP
CMP / GMP
LMPP / ELP
early MK
MK
Mast cell
early Ery
mid Ery
late Ery
Mono Mac
Dendritic cell
Myelocyte
ILC precursor
NK / T
early B cell
B cell

B

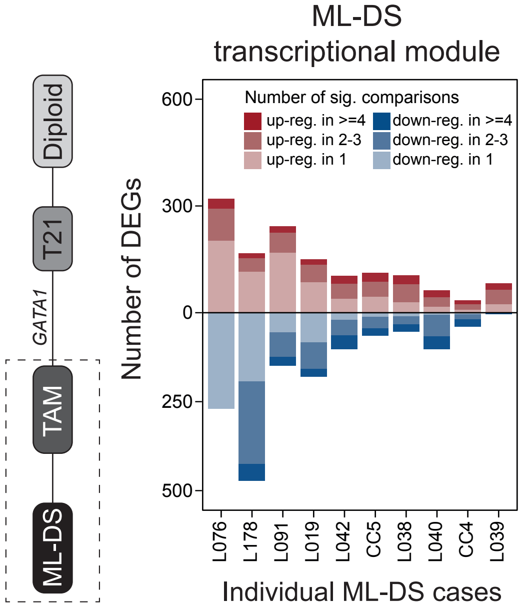
Diploid
T21
GATA1
TAM
ML-DS
ML-DS
transcriptional module
Number of sig. comparisons
up-reg. in >=4
up-reg. in 2-3
up-reg. in 1
down-reg. in >=4
down-reg. in 2-3
down-reg. in 1
Number of DEGs
600
300
0
250
500
L076
L178
L091
L019
L042
CC5
L038
L040
CC4
L039
Individual ML-DS cases

Supplementary Figure 5: Transcriptional changes underpinning TAM and ML-DS.

(A) Heatmap showing the z-scaled mean expression levels of the "*GATA1* – leukaemia" transcriptional module across haematopoietic lineages (columns) in our diploid fetal liver scRNA-seq. Darker red indicates higher expression. Only genes expressed in the diploid fetal liver are included. "early B cell" includes pro B cell and pre B cell; "dendritic cells" include dendritic cell 1, dendritic cell 2, and plasmacytoid dendritic cell. Genes encoding transcriptional factors are listed, genes located on chromosome 21 are listed and highlighted in purple. Black boxes highlight that genes upregulated in *GATA1*-mutant cells included many associated with megakaryocyte and mast cell lineages (1); whereas downregulated genes included many erythroid genes (2).

(B) Bar plot showing the number of differentially expressed genes (DEGs) detected when comparing diagnostic (treatment naive) blasts from each ML-DS case (x-axis) against all conventional TAM blasts. Genes are further grouped based on the number of comparisons that they were identified as significantly expressed in. Darker red indicates up-regulated genes and darker blue indicates down-regulated genes which are detected in multiple ML-DS cases when compared to conventional TAM.

Abbreviation

<u>Cell types</u>: CMP / GMP - common myeloid progenitor / granulocyte-monocyte progenitor;
Ery - erythroblast; HSC / MPP - haematopoietic stem cell / multipotent progenitor;
ILC precursor - innate lymphoid cell precursor;
LMPP / ELP - lymphoid-primed multipotent progenitor / early lymphoid progenitor;
MEMP / MEP - megakaryocyte-erythroid-mast cell progenitor / megakaryocyte-erythroid progenitor;
MK - megakaryocyte; Mono/Mac - monocyte / macrophage; NK / T - natural killer cell / T cell.

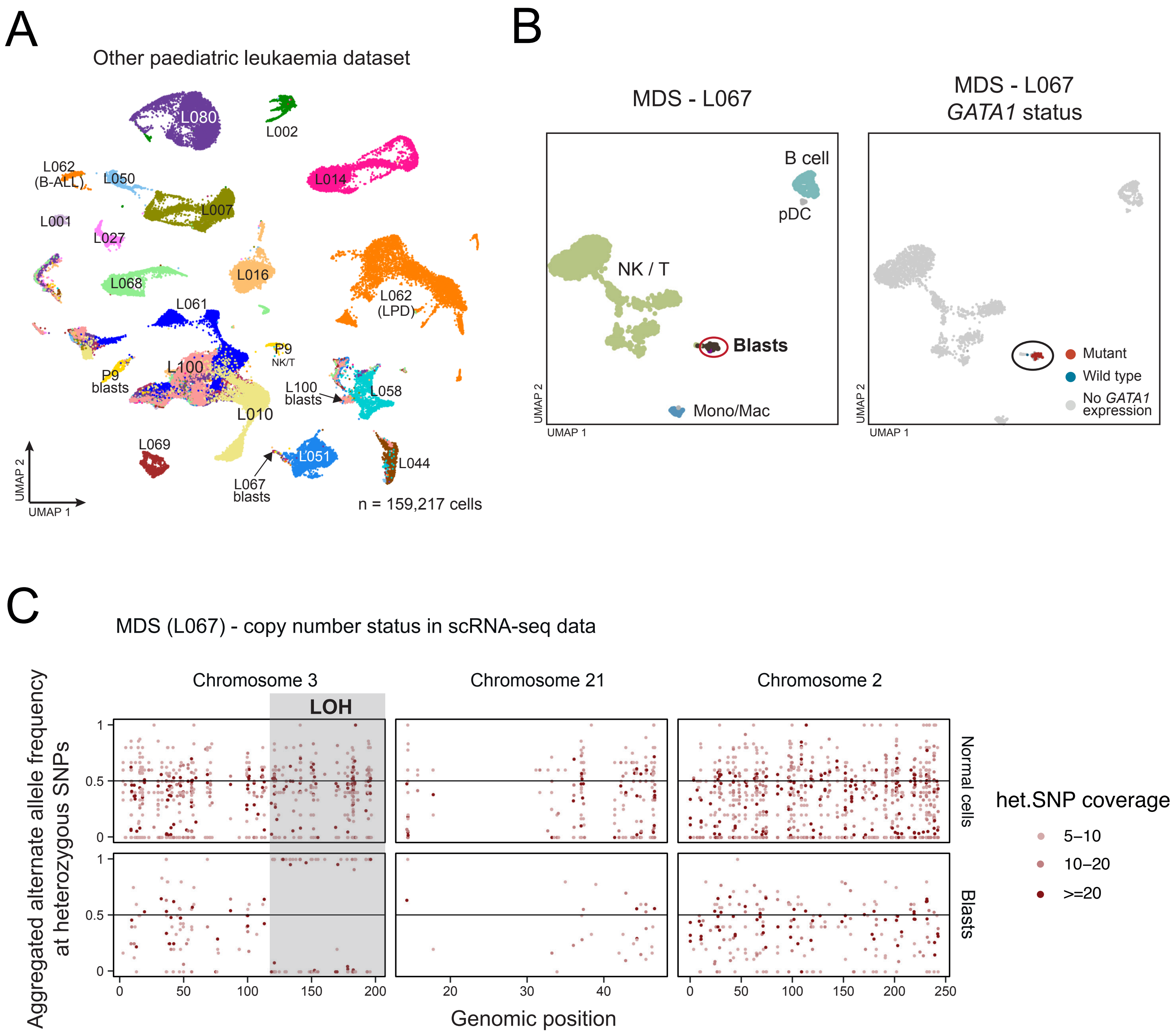
A
Other paediatric leukaemia dataset
L080
L002
L062 (B-ALL)
L050
L014
L007
L001
L027
L016
L068
L062 (LPD)
L061
P9 NK/T
P9 blasts
L100
L100 blasts
L058
L010
L069
L051
L044
L067 blasts
UMAP 2
UMAP 1
n = 159,217 cells
B
MDS - L067
B cell
pDC
NK / T
Blasts
Mono/Mac
MDS - L067
GATA1 status
Mutant
Wild type
No GATA1 expression
C
MDS (L067) - copy number status in scRNA-seq data
Chromosome 3
Chromosome 21
Chromosome 2
LOH
Normal cells
Blasts
Aggregated alternate allele frequency at heterozygous SNPs
Genomic position
het.SNP coverage
5–10
10–20
>=20

Supplementary Figure 6: Other leukaemias scRNA-seq dataset.

(A) UMAP visualisation of the additional scRNA-seq dataset of other leukaemias (detailed in Table 1, Supplementary Data 1), with cells (dots) coloured by donor ID. B-ALL - B cell acute lymphoblastic leukaemia; LPD - lymphoproliferative disorder.

(B) UMAP visualisation of the diploid myeloid neoplasm with *GATA1* mutation (MDS - myelodysplastic syndrome, donor L067). Cells (dots) are coloured by cell type (left) and the *GATA1* genotyping result (right). pDC - plasmacytoid dendritic cell, NK / T - natural killer cell / T cell, Mono/Mac - monocyte / macrophage.

(C) Scatter plots of the alternate-allele frequency (y-axis) for heterozygous single-nucleotide polymorphisms (SNPs) on chromosomes 3 (left), 21 (middle), and 2 (right) in scRNA-seq data of the MDS case (donor L067). The allele frequency is aggregated across normal cells (top) and blasts (bottom). Each dot represents a heterozygous SNP on the chromosome, coloured by the total coverage across all cells in the group. Grey-shaded region highlights the copy number loss on chromosome 3, where loss-of-heterozygosity is observed in blasts but not in normal cells. There is no evidence of a shift in alternate allele frequency across heterozygous SNPs on chromosome 21, thus no indication of copy number alteration on chromosome 21.

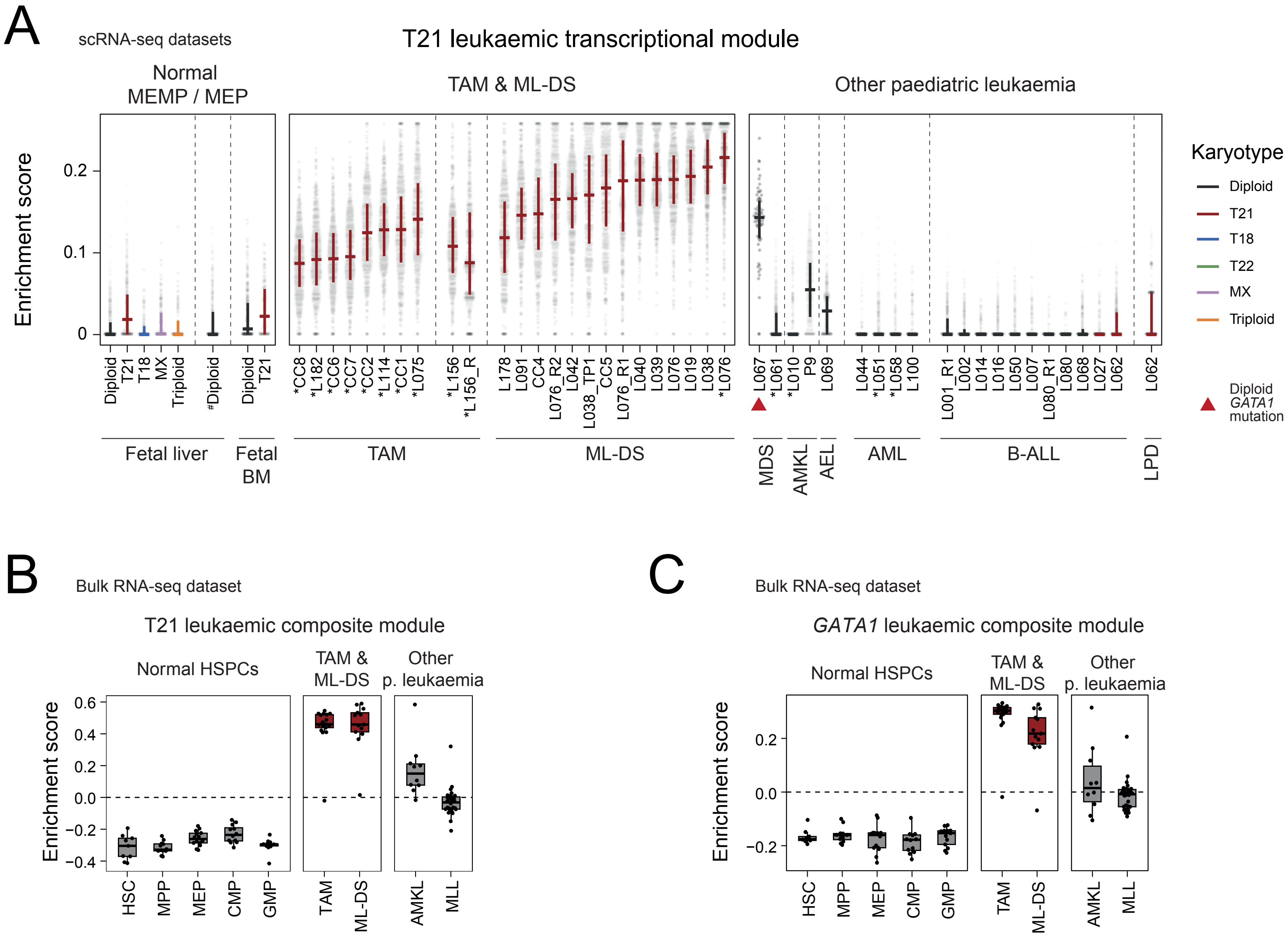
A
scRNA-seq datasets
T21 leukaemic transcriptional module
Normal MEMP / MEP
TAM & ML-DS
Other paediatric leukaemia
Enrichment score
0
0.1
0.2
Diploid
T21
T18
MX
Triploid
#Diploid
Diploid
T21
*CC8
*L182
*CC6
*CC7
*CC2
*L114
*CC1
*L075
*L156
*L156_R
L178
L091
CC4
L076_R2
L042
L038_TP1
CC5
L076_R1
L040
L039
L076
L019
L038
*L076
L067
*L061
*L010
P9
L069
L044
*L051
*L058
L100
L001_R1
L002
L014
L016
L050
L007
L080_R1
L080
L068
L027
L062
L062
Fetal liver
Fetal BM
TAM
ML-DS
MDS
AMKL
AEL
AML
B-ALL
LPD
Karyotype
Diploid
T21
T18
T22
MX
Triploid
Diploid GATA1 mutation
B
Bulk RNA-seq dataset
T21 leukaemic composite module
Normal HSPCs
TAM & ML-DS
Other p. leukaemia
Enrichment score
0.6
0.4
0.2
0.0
−0.2
−0.4
HSC
MPP
MEP
CMP
GMP
TAM
ML-DS
AMKL
MLL
C
Bulk RNA-seq dataset
GATA1 leukaemic composite module
Normal HSPCs
TAM & ML-DS
Other p. leukaemia
Enrichment score
0.2
0.0
−0.2
HSC
MPP
MEP
CMP
GMP
TAM
ML-DS
AMKL
MLL

Supplementary Figure 7: Specificity of the trisomy 21- and *GATA1* - leukaemia transcriptional modules.

(A) Enrichment score of the "trisomy 21 – leukaemia" transcriptional module (y-axis) across individual cells (grey dots) from various scRNA-seq datasets (x-axis) consisting of: normal MEMP/MEP with different karyotypes from the fetal liver (including diploid cells from Popescu, D-M. *et al.*, 2019[2], denoted by #) and fetal bone marrow (BM) from Jardine, L. *et al.*, 2021[3]; TAM/ML-DS blasts; and leukaemic blasts from other leukaemias. Leukaemic cells are grouped by patient, timepoint (initial diagnosis unless indicated otherwise in the group name) and tissue. Asterisks (*) indicate peripheral blood samples; all others are bone marrow aspirates. For each group, cross bar vertical line shows interquartile range, and horizontal line indicate the median enrichment score, coloured by donor germline karyotype.

(B) Enrichment score (y-axis) of the "trisomy 21 – leukaemia" transcriptional module in the independent bulk RNA-seq dataset composed of FACS-sorted normal haematopoietic stem and progenitor cells (HSPCs), TAM/ML-DS diagnostic samples, and leukaemic blasts from other paediatric leukaemias. Boxes indicate first and third quartiles, central line represents the median, and whiskers extend to 1.5× IQR. Boxes are coloured by sample karyotype using the same colour scheme as in panel (A).

(C) Enrichment score (y-axis) of the "*GATA1* – leukaemia" transcriptional module (top genes) in the independent bulk RNA-seq dataset as detailed in (B). Boxplots are defined as in (B), coloured by sample karyotype using the same colour scheme as in panel (A).

Abbreviation

Leukaemia: MDS - myelodysplastic syndrome; AMKL - acute megakaryoblastic leukaemia; AML - acute myeloid leukaemia; AEL - acute erythroid leukaemia; B-ALL - B cell acute lymphoblastic leukaemia; LPD - lymphoproliferative disorder; MLL - mixed lineage leukaemia.

Cell types: HSPCs - haematopoietic stem and progenitor cells; HSC - haematopoietic stem cell; MPP - multipotent progenitor; MEP - megakaryocyte-erythroid progenitor; CMP - common myeloid progenitor; GMP - granulocyte-monocyte progenitor; NK / T - natural killer cell / T cell.

Karyotypes: T - trisomy; MX - monosomy X.

Sample timepoint: R - recurrent; R1 - relapse 1 diagnosis; R2 - relapse 2 diagnosis; TP1 - timepoint 1.

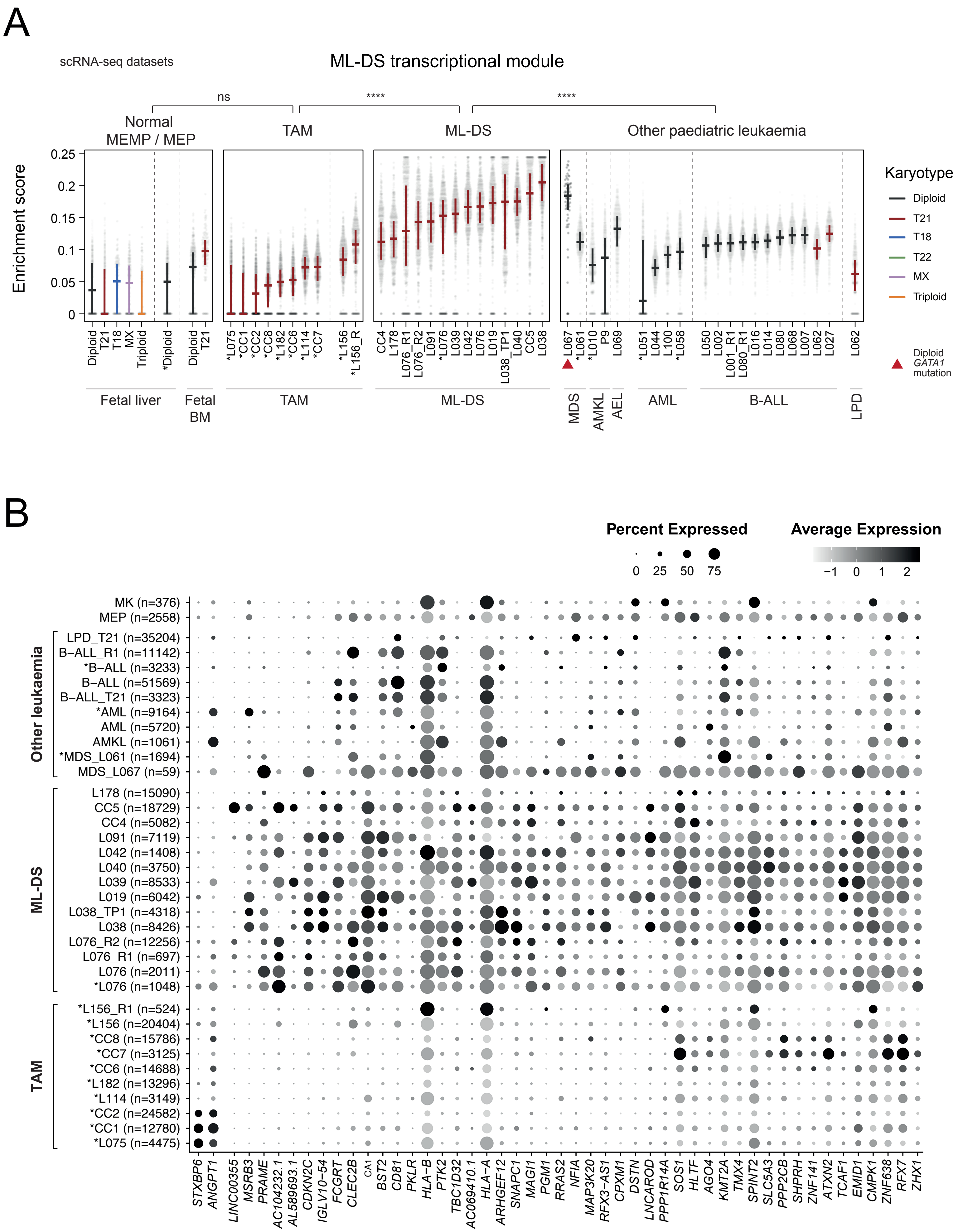

A
scRNA-seq datasets
ML-DS transcriptional module
ns
****
****
Normal MEMP / MEP
TAM
ML-DS
Other paediatric leukaemia
Enrichment score
0.25
0.2
0.15
0.1
0.05
0
Karyotype
Diploid
T21
T18
T22
MX
Triploid
Diploid GATA1 mutation
Diploid
T21
T18
MX
Triploid
#Diploid
Diploid
T21
*L075
*CC1
*CC2
*CC8
*L182
*CC6
*L114
*CC7
*L156
*L156_R
CC4
L178
L076_R1
L076_R2
L091
*L076
L039
L042
L076
L019
L038_TP1
L040
CC5
L038
L067
*L061
*L010
P9
L069
*L051
L044
L100
*L058
L050
L002
L001_R1
L080_R1
L016
L014
L080
L068
L007
L062
L027
L062
Fetal liver
Fetal BM
TAM
ML-DS
MDS
AMKL
AEL
AML
B-ALL
LPD
B
Percent Expressed
0
25
50
75
Average Expression
−1
0
1
2
MK (n=376)
MEP (n=2558)
Other leukaemia
LPD_T21 (n=35204)
B−ALL_R1 (n=11142)
*B−ALL (n=3233)
B−ALL (n=51569)
B−ALL_T21 (n=3323)
*AML (n=9164)
AML (n=5720)
AMKL (n=1061)
*MDS_L061 (n=1694)
MDS_L067 (n=59)
ML-DS
L178 (n=15090)
CC5 (n=18729)
CC4 (n=5082)
L091 (n=7119)
L042 (n=1408)
L040 (n=3750)
L039 (n=8533)
L019 (n=6042)
L038_TP1 (n=4318)
L038 (n=8426)
L076_R2 (n=12256)
L076_R1 (n=697)
L076 (n=2011)
*L076 (n=1048)
TAM
*L156_R1 (n=524)
*L156 (n=20404)
*CC8 (n=15786)
*CC7 (n=3125)
*CC6 (n=14688)
*L182 (n=13296)
*L114 (n=3149)
*CC2 (n=24582)
*CC1 (n=12780)
*L075 (n=4475)
STXBP6
ANGPT1
LINC00355
MSRB3
PRAME
AC104232.1
AL589693.1
CDKN2C
IGLV10−54
FCGRT
CLEC2B
CA1
BST2
CD81
PKLR
HLA−B
PTK2
TBC1D32
AC069410.1
HLA−A
ARHGEF12
SNAPC1
MAGI1
PGM1
RRAS2
NFIA
MAP3K20
RFX3−AS1
CPXM1
DSTN
LNCAROD
PPP1R14A
SOS1
HLTF
AGO4
KMT2A
TMX4
SPINT2
SLC5A3
PPP2CB
SHPRH
ZNF141
ATXN2
TCAF1
EMID1
CMPK1
ZNF638
RFX7
ZHX1

Supplementary Figure 8: Specificity of the ML-DS leukaemia transcriptional module.

(A) Enrichment score (y-axis) of the ML-DS transcriptional signature across individual cells (grey dots) from different scRNA-seq datasets (x-axis) consisting of: normal MEMP/MEP with different karyotypes from the fetal liver (including diploid cells from Popescu, D-M. *et al.*, 2019[2], denoted by #) and fetal bone marrow (BM) from Jardine, L. *et al.*, 2021[3]; TAM/ML-DS blasts; and leukaemic blasts from other leukaemias. Leukaemic cells are grouped by patient, timepoint (initial diagnosis unless indicated otherwise in the group name) and tissue. Asterisks (*) indicate peripheral blood samples; all others are bone marrow aspirates. For each group, cross bar vertical line shows interquartile range, and horizontal line indicate the median enrichment score, coloured by donor germline karyotype.
The per-sample median enrichment scores in ML-DS group (n=14 samples) are significantly higher than in TAM group (n=10 samples), and other leukaemia (n=21 samples) ($p < 0.0001$ for both comparisons, two-sided Wilcoxon rank sum test). No significant difference observed between TAM group (n=10 samples) and normal MEMP/MEP (n= 26 samples across three fetal datasets; $p > 0.05$, one-sided Wilcoxon rank sum test).

(B) Dot plot showing z-scaled mean expression levels (colour) of top up- and down-regulated genes in ML-DS diagnostic blasts compared to conventional TAM blasts (i.e. the ML-DS leukaemic gene module). Dot size represents the proportion of cells with positive expression. Leukaemic cells are grouped by subtype, patient, timepoint (initial diagnosis unless indicated otherwise in the group name) and tissue. Asterisks (*) indicate peripheral blood samples; all others are bone marrow aspirates. Normal megakaryocyte-erythrocyte progenitors (MEP) and megakaryocytes (MK) obtained from both leukaemia datasets are also shown. The number of cells in each group is indicated inside brackets (the *n* values).

Abbreviation
Cell types: MEP - megakaryocyte-erythroid progenitor; MK - megakaryocyte.
Leukaemia: MDS - myelodysplastic syndrome; AMKL - acute megakaryoblastic leukaemia;
AML - acute myeloid leukaemia; AEL - acute erythroid leukaemia;
B-ALL - B cell acute lymphoblastic leukaemia; LPD - lymphoproliferative disorder.
Sample timepoint: R - recurrent; R1 - relapse 1 diagnosis; R2 - relapse 2 diagnosis; TP1 - timepoint 1.
Karyotypes: T - trisomy; MX - monosomy X.

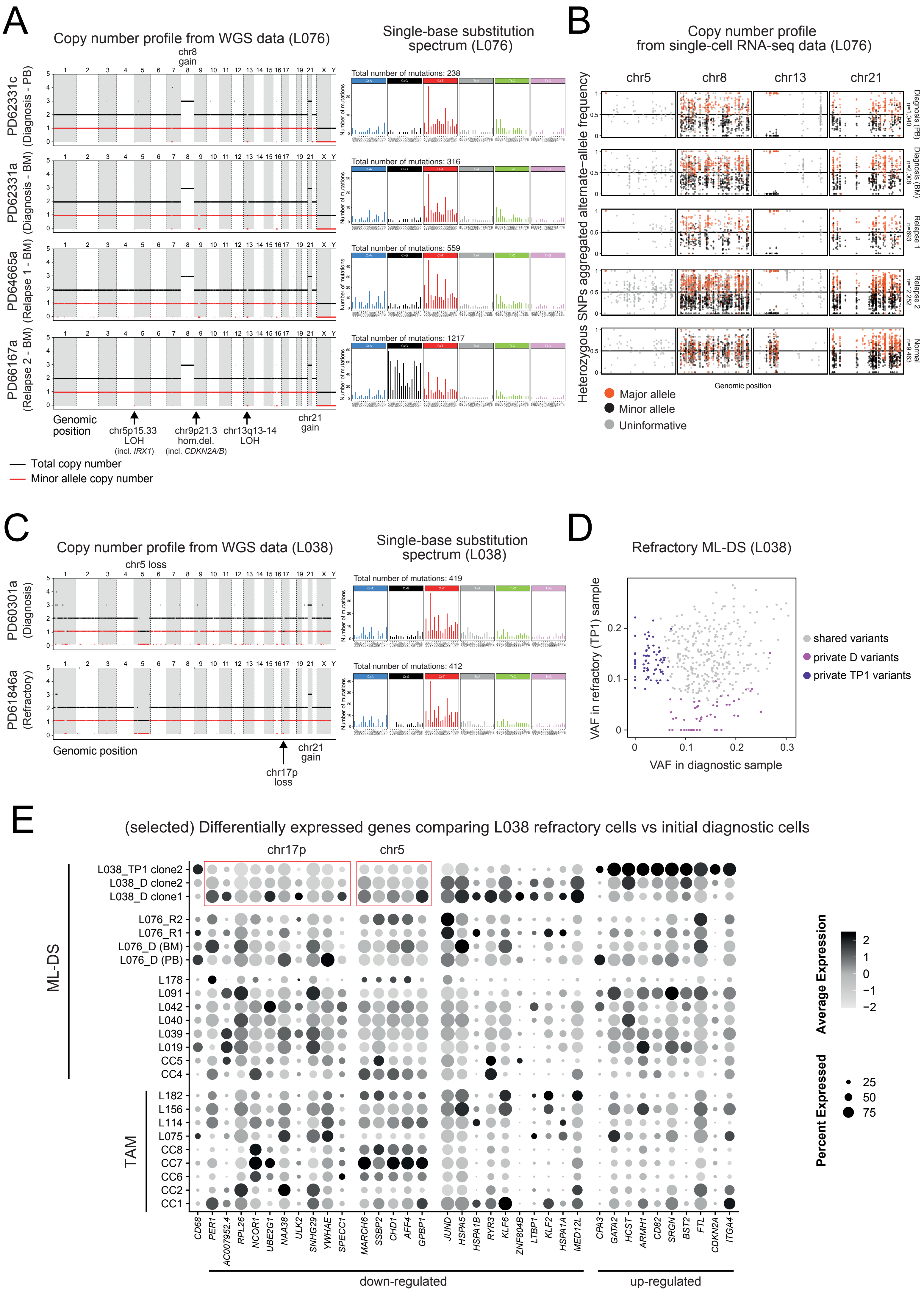
A
Copy number profile from WGS data (L076)
chr8 gain
PD62331c (Diagnosis - PB)
PD62331a (Diagnosis - BM)
PD64665a (Relapse 1 - BM)
PD66167a (Relapse 2 - BM)
Genomic position
chr5p15.33 LOH (incl. IRX1)
chr9p21.3 hom.del. (incl. CDKN2A/B)
chr13q13-14 LOH
chr21 gain
Total copy number
Minor allele copy number
Single-base substitution spectrum (L076)
Total number of mutations: 238
Total number of mutations: 316
Total number of mutations: 559
Total number of mutations: 1217
Number of mutations
C>A
C>G
C>T
T>A
T>C
T>G
B
Copy number profile from single-cell RNA-seq data (L076)
chr5
chr8
chr13
chr21
Heterozygous SNPs aggregated alternate-allele frequency
Diagnosis (PB) n=1,040
Diagnosis (BM) n=2,008
Relapse 1 n=693
Relapse 2 n=12,252
Normal n=9,463
Genomic position
Major allele
Minor allele
Uninformative
C
Copy number profile from WGS data (L038)
chr5 loss
PD60301a (Diagnosis)
PD61846a (Refractory)
Genomic position
chr21 gain
chr17p loss
Single-base substitution spectrum (L038)
Total number of mutations: 419
Total number of mutations: 412
D
Refractory ML-DS (L038)
VAF in refractory (TP1) sample
VAF in diagnostic sample
shared variants
private D variants
private TP1 variants
E
(selected) Differentially expressed genes comparing L038 refractory cells vs initial diagnostic cells
chr17p
chr5
ML-DS
L038_TP1 clone2
L038_D clone2
L038_D clone1
L076_R2
L076_R1
L076_D (BM)
L076_D (PB)
L178
L091
L042
L040
L039
L019
CC5
CC4
TAM
L182
L156
L114
L075
CC8
CC7
CC6
CC2
CC1
CD68
PER1
AC007952.4
RPL26
NCOR1
UBE2G1
NAA38
ULK2
SNHG29
YWHAE
SPECC1
MARCH6
SSBP2
CHD1
AFF4
GPBP1
JUND
HSPA5
HSPA1B
RYR3
KLF6
ZNF804B
LTBP1
KLF2
HSPA1A
MED12L
CPA3
GATA2
HCST
ARMH1
CD82
SRGN
BST2
FTL
CDKN2A
ITGA4
down-regulated
up-regulated
Average Expression
Percent Expressed
25
50
75

Supplementary Figure 9: The genetic and transcriptional evolution of progressive ML-DS.

(A) Copy number profiles derived from whole genome sequencing (WGS) data of four samples from child L076 with two relapses after initial treatment for ML-DS; as called by HMF-PURPLE[4]. Note that PURPLE[4] is unable to determine subclonal copy number aberrations. For each chromosome, the black line indicates the total copy number, and the red line indicates the minor allele copy number. All samples showed gain of chromosome 21 and chromosome 8, gain, loss of heterozygosity (LOH) on chromosome 13q and at 15p15.33, and a focal homozygous deletion at chromosome 9p21.3.

(B) Copy number genotyping in scRNA-seq data from the same samples as in (A) from child L076. Cells are grouped by identity (normal or cancer) and sample timepoint (rows). The aggregated B-allele frequency (BAF) (y-axis) of heterozygous single-nucleotide polymorphisms (SNPs) across chromosomes 5, 8, 13 and 21 (columns) is shown for each cell category. Each dot represents a heterozygous SNP. Orange dots indicate alternate alleles located on the major chromosome, whereas black dots represent alternate alleles located on the minor chromosome. Grey dots are heterozygous SNPs with insufficient evidence of BAF deviation from the expected value of 0.5. Unlike normal cells, cancer cells from all timepoints exhibit deviation in BAF of heterozygous SNPs on chromosome 8 and 21, as well as chromosome 13q (loss of heterozygosity). This confirms the identity of the cancer cells, and suggests that these copy number alterations are early events, thus pervading all cancer cells.

(C) Copy number profiles from WGS data of two samples from child L038 with refractory ML-DS; analysed using HMF-PURPLE[4]. Note that PURPLE is unable to determine whether the copy number aberrations are subclonal. For each chromosome, the black line indicates the total copy number, and the red line indicates the minor allele copy number. All samples showed chromosome 21 gain and 17p loss. The diagnostic sample showed evidence of chromosome 5q loss, whereas the timepoint 1 sample showed loss of the entire chromosome 5.

(D) Scatter plot showing the observed variant allele frequency (VAF) distribution from WGS data of two samples from child L038: diagnostic sample on x-axis and TP1 sample on y-axis. D - Diagnostic; TP1 - timepoint 1 (post 1 course of chemotherapy).

(E) Dot plot showing the average expression of top differentially expressed genes in L038 clone 2 from TP1 sample (with chr17p loss, including loss of *TP53*) compared to clone 1 from diagnostic sample (without chr17p loss). Dot size represents the percentage of cells expressing each gene; colour shows the z-scaled normalised expression levels, where darker grey indicates higher expression.

Abbreviation

Chr - chromosome

<u>Sample timepoint</u>: D - diagnosis; R1 - relapse diagnosis; R2 - relapse 2 diagnosis; TP1 - timepoint 1.

<u>Tissue</u>: PB - peripheral blood; BM - bone marrow.

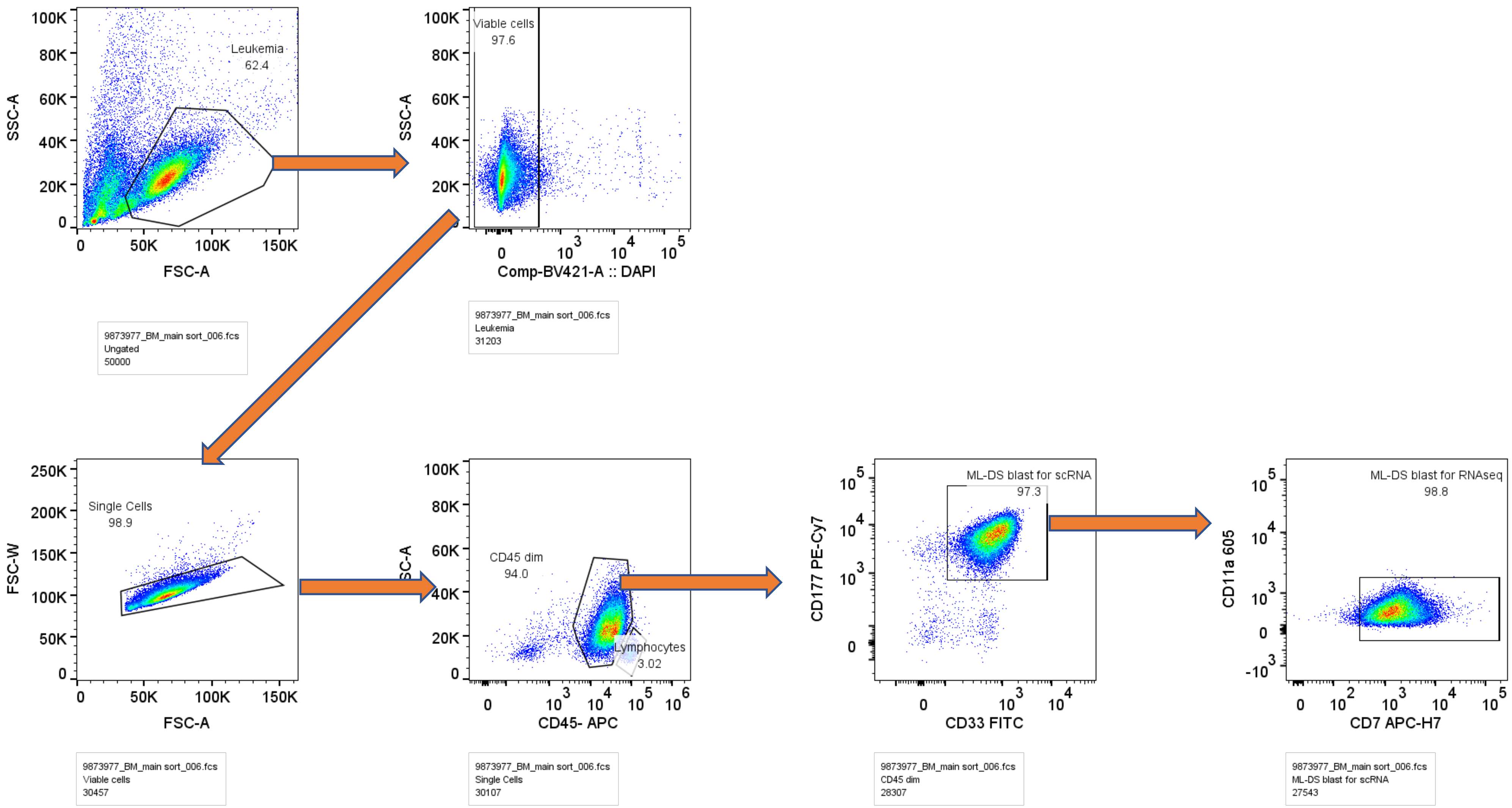

Leukemia
62.4
SSC-A
FSC-A
9873977_BM_main sort_006.fcs
Ungated
50000
Viable cells
97.6
SSC-A
Comp-BV421-A :: DAPI
9873977_BM_main sort_006.fcs
Leukemia
31203
Single Cells
98.9
FSC-W
FSC-A
9873977_BM_main sort_006.fcs
Viable cells
30457
CD45 dim
94.0
Lymphocytes
3.02
CD45- APC
9873977_BM_main sort_006.fcs
Single Cells
30107
ML-DS blast for scRNA
97.3
CD177 PE-Cy7
CD33 FITC
9873977_BM_main sort_006.fcs
CD45 dim
28307
ML-DS blast for RNAseq
98.8
CD11a 605
CD7 APC-H7
9873977_BM_main sort_006.fcs
ML-DS blast for scRNA
27543

Supplementary Figure 10: Representative gating strategy for isolation of leukaemic blasts.

TAM and ML-DS patient samples from the AML Berlin-Frankfurt-Münster study group were processed by flow cytometry to obtain purified leukaemic blasts for downstream 10x scRNA-seq.

Supplementary References

1. Domínguez Conde, C. *et al.* Cross-tissue immune cell analysis reveals tissue-specific features in humans. *Science* **376**, eabl5197 (2022).

2. Popescu, D.-M. *et al.* Decoding human fetal liver haematopoiesis. *Nature* **574**, 365–371 (2019).

3. Jardine, L. *et al.* Blood and immune development in human fetal bone marrow and Down syndrome. *Nature* **598**, 327–331 (2021).

4. Priestley, P. *et al.* Pan-cancer whole-genome analyses of metastatic solid tumours. *Nature* **575**, 210–216 (2019).
