# Supplementary material for: Single cell transcriptional evolution of myeloid leukemia of Down syndrome
Source: Nat Commun. 2026 Apr 23;17:3474. doi: 10.1038/s41467-026-71707-2 (PMC13106683; doi:10.1038/s41467-026-71707-2)
Supplement: Supplementary file 2 — Description of Additional Supplementary Files [file 41467_2026_71707_MOESM2_ESM.pdf]

Description of Additional Supplementary Files

Supplementary Data 1: Paediatric leukaemia and fetal liver datasets overview.

Supplementary Data 2: Differential gene expression analysis comparing cells from fetuses with abnormal karyotypes against diploid cells, for each cell type and each karyotype. Differential expression was assessed using a two-sided Wald test in DESeq21 with Benjamini-Hochberg correction for multiple hypothesis testing.

Supplementary Data 3: List of differentially expressed genes representing the contribution of trisomy 21 towards leukaemic transcriptome. Differential expression was assessed using a two-sided Wald test in DESeq21 with Benjamini-Hochberg correction for multiple hypothesis testing.

Supplementary Data 4: List of differentially expressed genes representing the transcriptomic consequences of GATA1 mutations towards leukaemic transcriptome. Differential expression was assessed using a two-sided Wald test in DESeq21 with Benjamini-Hochberg correction for multiple hypothesis testing.

Supplementary Data 5: List of differentially expressed genes representing the transcriptomic differences between conventional TAM blasts and ML-DS diagnostic bone marrow blasts. Differential expression was assessed using a two-sided Wald test in DESeq21 with Benjamini-Hochberg correction for multiple hypothesis testing.

Supplementary Data 6: Catalogue of somatic mutations in L076 with relapse ML-DS.

Supplementary Data 7: Catalogue of somatic mutations in L038 with refractory ML-DS.

Supplementary Data 8: List of markers when comparing progressive ML-DS blasts against diagnostic ML-DS blasts of L076 and L038. Marker detection was performed using the two-sided Wilcoxon ranksum test implemented in the FindMarkers function from the R package Seurat2, with Bonferroni correction for multiple hypothesis testing.

Supplementary Data 9: Bulk RNA-seq count matrix. Raw gene-level count table for bulk RNA-seq samples, including FACS-sorted normal HSPCs, TAM blasts, ML-DS blasts, and additional paediatric leukaemia samples (AMKL and MLL). Gene expression was quantified using the STAR3/Salmon4 workflow. Each column represents an individual biological sample; each row corresponds to a gene. This file contains the un-normalised counts used for all bulk RNA-seq analyses in the manuscript. Sample-level metadata are provided in Supplementary Data 1.
